# Crystal structure of benzyl (*E*)-2-(3,4-di­meth­oxy­benzyl­idene)hydrazine-1-carbodi­thio­ate

**DOI:** 10.1107/S205698901500095X

**Published:** 2015-01-31

**Authors:** Yew-Fung Tan, Mohammed Khaled bin Break, M. Ibrahim M. Tahir, Teng-Jin Khoo

**Affiliations:** a Centre for Natural and Medicinal Product Research, School of Pharmacy, University of Nottingham Malaysia Campus, 43500 Semenyih, Malaysia; b Department of Chemistry, Faculty of Science, Universiti Putra, Malaysia

**Keywords:** crystal structure, di­thio­carbazate, hydrazine-1-carbodi­thio­ate, 3,4-di­meth­oxy­benzaldehyde, hydrogen bonding, C—H⋯π inter­actions

## Abstract

The title compound, C_17_H_18_N_2_O_2_S_2_, synthesized *via* a condensation reaction between *S*-benzyl di­thio­carbazate and 3,4-di­meth­oxy­benzaldehyde, crystallized with two independent mol­ecules (*A* and *B*) in the asymmetric unit. Both mol­ecules have an L-shape but differ in the orientation of the benzyl ring with respect to the 3,4-di­meth­oxy­benzyl­idine ring, this dihedral angle is 65.59 (8)° in mol­ecule *A* and 73.10 (8)° in mol­ecule *B*. In the crystal, the *A* and *B* mol­ecules are linked *via* pairs of N—H⋯S hydrogen bonds, forming dimers with an *R*_2_^2^(8) ring motif. The dimers are linked *via* pairs of C—H⋯O hydrogen bonds, giving inversion dimers of dimers. These units are linked by C—H⋯π inter­actions, forming ribbons propagating in the [100] direction.

## Abstract 2

In the crystal of the title compound, which crystallized with two independent mol­ecules (*A* and *B*) in the asymmetric unit, the *A* and *B* mol­ecules are linked *via* pairs of N—H⋯S hydrogen bonds, forming dimers with an 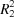
(8) ring motif. The dimers are linked *via* pairs of C—H⋯O hydrogen bonds and C—H⋯π inter­actions, forming ribbons propagating along [100].

## Chemical context   

Schiff bases have been proven to possess a variety of bio­logical activities, and this has led to extensive studies on this group of compounds with particular emphasis on those derived from di­thio­carbaza­tes. Di­thio­carbazate-derived Schiff bases have generally been found to exhibit inter­esting cytotoxic and anti­microbial activities. One of the most investigated di­thio­carbaza­tes has been *S-*benzyl­dithio­carbazate (SBDTC) whose derivatives have shown promising biological activities (Break *et al.*, 2013 ▸). Therefore, as part of our research which is aimed at developing anti­cancer and anti­microbial drugs, we have synthesized a novel Schiff base *via* the condensation reaction of SBDTC and 3,4-di­meth­oxy­benzaldehyde. We report herein on the synthesis and crystal structure of the title compound.
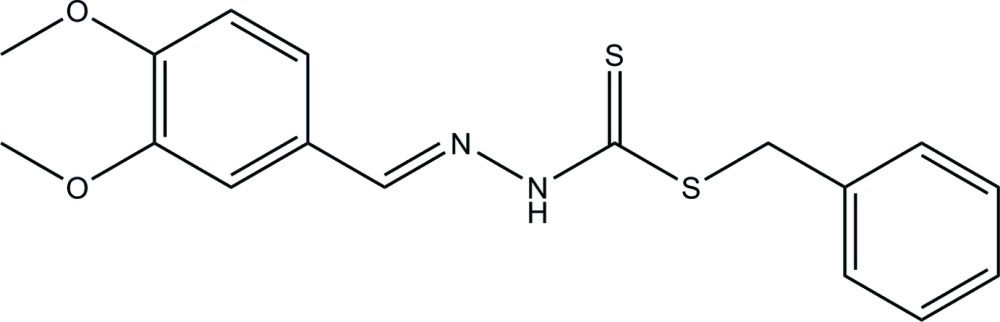


## Structural commentary   

**Figure 1:**
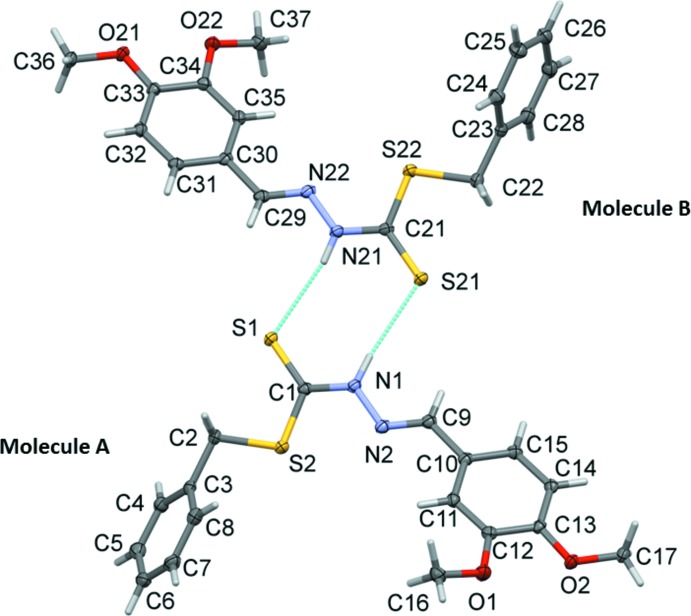
A view of the mol­ecular structure of the two independent mol­ecules (*A* and *B*) of the title compound, showing the atom labelling. Displacement ellipsoids are drawn at the 50% probability level. N—H⋯S hydrogen bonds are shown as dashed lines (see Table 1 ▸ for details).

**Figure 2:**
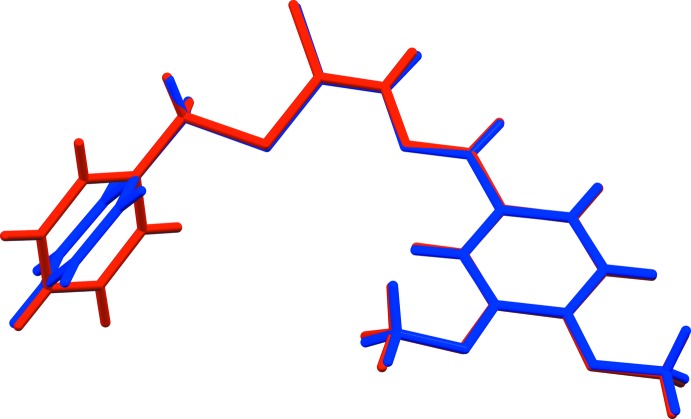
A view of the mol­ecular overlay (Mercury; Macrae *et al.*, 2008 ▸) of the two independent mol­ecules (*A* blue and *B* red) of the title compound.

The title compound, Fig. 1 ▸, crystallized with two independent mol­ecules (*A* and *B*) in the asymmetric unit. Both mol­ecules have an l-shape but differ in the orientation of the benzyl ring with respect to the 3,4-di­meth­oxy­benzyl­idine ring, this dihedral angle being 65.59 (8) ° in mol­ecule *A* and 73.10 (8) ° in mol­ecule *B* (Fig. 2 ▸).

The C—N and N(H)—C bond lengths (C1—N1 and N2—C9 in *A*, and C21—N21 and N22—C29 in *B*) are 1.331 (2) and 1.282 (2) Å, respectively, in mol­ecule *A*, and 1.336 (2) and 1.280 (2) Å, respectively, in mol­ecule *B*. The shorter length of the C—N bond suggests the existence of a double bond which belongs to the imine group. Similarly, the shorter C—S bond length [C1—S1 = 1.681 (2) Å in *A*, and C21—S21 = 1.677 (2) Å in *B*] relative to that of [C1—S2 = 1.749 (2) Å in *A*, and C21—S22 = 1.749 (2) Å in *B*] suggests that the former possesses double-bond character, indicating that the mol­ecule exists in its thione form in the solid state. The functional group identities proposed from these bond lengths are further supported by data obtained from the IR analysis reported below. Furthermore, the bond distances in the title compound are similar to those found for other carbodi­thio­ate-derived Schiff bases (Break *et al.*; 2013 ▸; Khoo *et al.*, 2014 ▸).

Both mol­ecules (*A* and *B*) crystallizes in the conformer in which the two aromatic rings of the compound are *cis* with respect to each other across the C=N bonds, while the thione sulfur atom is *trans* with respect to the same bond.

## Supra­molecular features   

**Figure 3:**
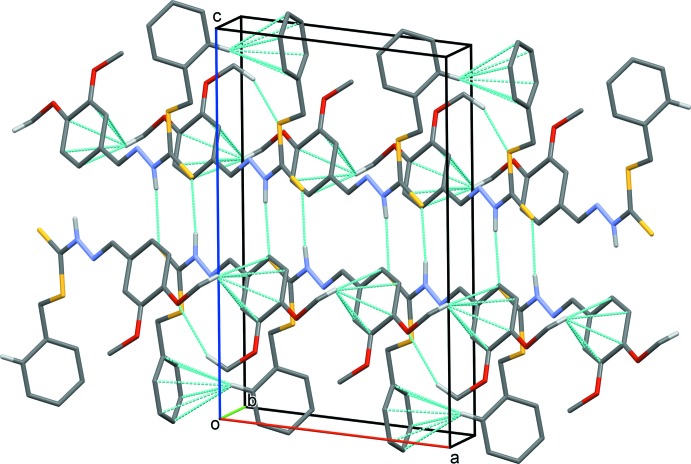
A view approximately along the *b* axis of the crystal structure of the title compound. The hydrogen bonds and C—H⋯π inter­actions are shown as dashed lines (see Table 1 ▸ for details; for clarity only the H atoms involved in these inter­actions are shown).

In the crystal, the *A* and *B* mol­ecules are linked by pairs of N—H⋯S hydrogen bonds, forming dimers with an *R_2_^2^* (8) ring motif (Table 1 ▸ and Fig. 3 ▸). The dimers are linked *via* pairs of C—H⋯O hydrogen bonds, giving inversion dimers of dimers. These units are linked by C—H⋯π inter­actions, forming ribbons propagating in the [100] direction (Fig. 3 ▸ and Table 1 ▸).

## Database survey   

A search of the Cambridge Structural Database (Version 5.35, May 2014; Groom & Allen, 2014 ▸) for benzyl (*E*)-2-benzyl­idenehydrazine-1-carbodi­thio­ates gave 13 hits. One of these concerns a structure very similar to the title compound, namely benzyl (*E*)-2-(4-meth­oxy­benzyl­idene)hydrazine-1-carbodi­thio­ate (YAHDAO; Fan *et al.*, 2011 ▸). Here the two aromatic rings are inclined to one another by *ca* 85.71°, compared with 65.59 (8)° in mol­ecule *A* and 73.10 (8)° in mol­ecule *B* of the title compound.

## Synthesis and crystallization   

1.98 g (0.01 mol) of *S*-benzyl­dithio­carbazate in 30 ml of absolute ethanol was added to an equimolar qu­antity of 3,4-di­meth­oxy­benzaldehyde in 10 ml of absolute ethanol, followed by the addition of 2–4 drops of concentrated H_2_SO_4_. The mixture was heated over a steam bath for 15 min and a precipitate started to form. The Schiff base which precipitated was filtered, washed with cold ethanol and dried *in vacuo* over silica gel, giving a white yellowish product. Yellow crystals of the title compound, suitable for X-ray analysis, were obtained by slow evaporation of a solution in DMSO over a period of three weeks (yield 60%; m.p. 438–439 K). IR (KBr, cm^−1^): 3360, 3122, 1602, 1069, 1023, 950, 788, 695. LCMS (ESI^+^): 347.1 [*M*+H]^+^.

## Refinement   

Crystal data, data collection and structure refinement details are summarized in Table 2 ▸. The H atoms were all located in a difference Fourier map, but those attached to carbon atoms were repositioned geometrically. The H atoms were initially refined with soft restraints on the bond lengths and angles to regularize their geometry (C—H in the range 0.93–0.98 Å, N—H in the range 0.86–0.89 Å), with *U*_iso_(H) = 1.5*U*_eq_(C) for methyl H atoms and = 1.2*U*_eq_(C) for other H atoms.

## Supplementary Material

Crystal structure: contains datablock(s) I, global. DOI: 10.1107/S205698901500095X/su5044sup1.cif

Structure factors: contains datablock(s) I. DOI: 10.1107/S205698901500095X/su5044Isup2.hkl

Supporting information file. DOI: 10.1107/S205698901500095X/su5044Isup3.cml

CCDC reference: 1043887

Additional supporting information:  crystallographic information; 3D view; checkCIF report

## Figures and Tables

**Table 1:** Hydrogen-bond geometry (, ) *Cg*1, *Cg*2 and *Cg*4 are the centroids of the C3C8, C10C15 and C30C35 rings, respectively.

*D*H*A*	*D*H	H*A*	*D* *A*	*D*H*A*
N1H2S21	0.92	2.52	3.418(1)	166
N21H1S1	0.87	2.55	3.407(1)	168
C16H163O21^i^	0.97	2.69	3.560(2)	149
C17H172*Cg*4^ii^	0.97	2.74	3.5587(18)	143
C28H281*Cg*1^ii^	0.94	2.94	3.6082(18)	130
C36H363*Cg*2^i^	0.97	2.67	3.5023(17)	145

Symmetry codes: (i) 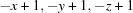; (ii) 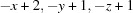.

**Table 2:** Experimental details

Crystal data	
Chemical formula	C_17_H_18_N_2_O_2_S_2_
*M*_r_	346.45
Crystal system, space group	Triclinic, *P* 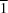
Temperature (K)	100
*a*, *b*, *c* ()	9.6432(5), 10.796(1), 16.1673(10)
, , ()	90.899(6), 97.203(5), 91.200(6)
*V* (^3^)	1669.2(2)
*Z*	4
Radiation type	Cu *K*
(mm^1^)	2.98
Crystal size (mm)	0.15 0.06 0.04

Data collection	
Diffractometer	Oxford Diffraction Gemini
Absorption correction	Multi-scan (*CrysAlis RED*; Oxford Diffraction, 2002 ▸)
*T*_min_, *T*_max_	0.74, 0.89
No. of measured, independent and observed [*I* > 2(*I*)] reflections	23678, 6592, 5514
*R*_int_	0.028
(sin /)_max_ (^1^)	0.622

Refinement	
*R*[*F* ^2^ > 2(*F* ^2^)], *wR*(*F* ^2^), *S*	0.037, 0.106, 0.99
No. of reflections	6567
No. of parameters	415
H-atom treatment	H-atom parameters constrained
_max_, _min_ (e ^3^)	0.44, 0.33

Computer programs: *CrysAlis CCD* and *CrysAlis RED* (Oxford Diffraction, 2002 ▸), *SUPERFLIP* (Palatinus Chapuis, 2007 ▸), *CRYSTALS* (Betteridge *et al.*, 2003 ▸), *Mercury* (Macrae *et al.*, 2008 ▸) and *publCIF* (Westrip, 2010 ▸).

## supplementary crystallographic information

### Crystal data

**Table:**

C_17_H_18_N_2_O_2_S_2_	*Z* = 4
*M**_r_* = 346.45	*F*(000) = 728
Triclinic, *P*1	*D*_x_ = 1.379 Mg m^−^^3^
Hall symbol: -P 1	Cu *K*α radiation, λ = 1.54180 Å
*a* = 9.6432 (5) Å	Cell parameters from 8676 reflections
*b* = 10.796 (1) Å	θ = 4–73°
*c* = 16.1673 (10) Å	µ = 2.98 mm^−^^1^
α = 90.899 (6)°	*T* = 100 K
β = 97.203 (5)°	Plate, yellow
γ = 91.200 (6)°	0.15 × 0.06 × 0.04 mm
*V* = 1669.2 (2) Å^3^	

### Data collection

**Table:**

Oxford Diffraction Gemini diffractometer	5514 reflections with *I* > 2σ(*I*)
Graphite monochromator	*R*_int_ = 0.028
ω scans	θ_max_ = 73.4°, θ_min_ = 4.1°
Absorption correction: multi-scan (*CrysAlis RED*; Oxford Diffraction, 2002)	*h* = −11→11
*T*_min_ = 0.74, *T*_max_ = 0.89	*k* = −13→11
23678 measured reflections	*l* = −20→20
6592 independent reflections	

### Refinement

**Table:**

Refinement on *F*^2^	Primary atom site location: other
Least-squares matrix: full	Hydrogen site location: difference Fourier map
*R*[*F*^2^ > 2σ(*F*^2^)] = 0.037	H-atom parameters constrained
*wR*(*F*^2^) = 0.106	Method = Modified Sheldrick *w* = 1/[σ^2^(*F*^2^) + ( 0.07*P*)^2^ + 0.48*P*] , where *P* = (max(*F*_o_^2^,0) + 2*F*_c_^2^)/3
*S* = 0.99	(Δ/σ)_max_ = 0.001
6567 reflections	Δρ_max_ = 0.44 e Å^−^^3^
415 parameters	Δρ_min_ = −0.33 e Å^−^^3^
0 restraints	

### Special details

**Table:**

Experimental. The crystal was placed in the cold stream of an Oxford Cryosystems open-flow nitrogen cryostat (Cosier & Glazer, 1986) with a nominal stability of 0.1K. Cosier, J. & Glazer, A.M., 1986. J. Appl. Cryst. 105-107.

### Fractional atomic coordinates and isotropic or equivalent isotropic displacement parameters (Å^2^)

**Table:**

	*x*	*y*	*z*	*U*_iso_*/*U*_eq_	
S1	0.67401 (4)	0.61670 (4)	0.39922 (2)	0.0170	
C1	0.77157 (15)	0.52035 (14)	0.34926 (9)	0.0150	
S2	0.78189 (4)	0.51826 (4)	0.24198 (2)	0.0182	
C2	0.66277 (16)	0.64058 (15)	0.20558 (10)	0.0187	
C3	0.68192 (15)	0.66561 (15)	0.11569 (9)	0.0174	
C8	0.65456 (17)	0.57319 (16)	0.05415 (10)	0.0215	
C7	0.67331 (17)	0.59709 (17)	−0.02786 (10)	0.0245	
C6	0.71768 (17)	0.71377 (18)	−0.04941 (10)	0.0243	
C5	0.74303 (17)	0.80681 (17)	0.01141 (10)	0.0239	
C4	0.72699 (16)	0.78255 (16)	0.09388 (10)	0.0199	
N1	0.85201 (13)	0.43802 (12)	0.39143 (8)	0.0163	
N2	0.92928 (13)	0.35596 (12)	0.35025 (8)	0.0171	
C9	1.01861 (16)	0.29639 (15)	0.39886 (10)	0.0171	
C10	1.10446 (16)	0.20035 (14)	0.36811 (9)	0.0156	
C15	1.20578 (16)	0.14725 (15)	0.42490 (10)	0.0167	
C14	1.28967 (15)	0.05382 (15)	0.39928 (9)	0.0159	
C13	1.27152 (15)	0.01212 (14)	0.31724 (10)	0.0154	
O2	1.34580 (11)	−0.07889 (11)	0.28523 (7)	0.0187	
C17	1.44520 (17)	−0.14133 (16)	0.34241 (10)	0.0204	
C12	1.16634 (16)	0.06392 (15)	0.25933 (9)	0.0168	
C11	1.08539 (16)	0.15789 (15)	0.28466 (10)	0.0169	
O1	1.15407 (12)	0.01059 (11)	0.18189 (7)	0.0235	
C16	1.03032 (18)	0.03484 (18)	0.12691 (10)	0.0242	
S21	0.84603 (4)	0.37456 (4)	0.59742 (2)	0.0167	
C21	0.74525 (15)	0.46870 (14)	0.64640 (9)	0.0153	
S22	0.73586 (4)	0.47065 (4)	0.75377 (2)	0.0186	
C22	0.84857 (16)	0.34265 (15)	0.78893 (10)	0.0186	
C23	0.82688 (16)	0.31752 (15)	0.87789 (10)	0.0177	
C28	0.92759 (17)	0.35307 (16)	0.94417 (10)	0.0218	
C27	0.90523 (18)	0.32945 (16)	1.02578 (10)	0.0245	
C26	0.78224 (19)	0.27209 (16)	1.04222 (10)	0.0241	
C25	0.68103 (18)	0.23642 (17)	0.97647 (10)	0.0243	
C24	0.70366 (17)	0.25821 (16)	0.89488 (10)	0.0219	
N21	0.66212 (13)	0.54892 (12)	0.60336 (8)	0.0167	
N22	0.58326 (14)	0.63039 (12)	0.64363 (8)	0.0174	
C29	0.49371 (16)	0.68920 (15)	0.59483 (10)	0.0165	
C30	0.40710 (15)	0.78438 (14)	0.62621 (10)	0.0156	
C31	0.30524 (16)	0.83952 (15)	0.57104 (9)	0.0160	
C32	0.22248 (15)	0.93261 (15)	0.59875 (9)	0.0161	
C33	0.24252 (15)	0.97166 (14)	0.68106 (9)	0.0154	
O21	0.17151 (11)	1.06326 (10)	0.71518 (7)	0.0186	
C36	0.07068 (16)	1.12784 (15)	0.65980 (10)	0.0198	
C34	0.34780 (16)	0.91696 (15)	0.73749 (9)	0.0165	
C35	0.42769 (16)	0.82408 (15)	0.71021 (9)	0.0169	
O22	0.36178 (12)	0.96604 (11)	0.81611 (7)	0.0226	
C37	0.48390 (17)	0.93340 (17)	0.86989 (10)	0.0229	
H21	0.6844	0.7164	0.2396	0.0224*	
H22	0.5671	0.6108	0.2095	0.0226*	
H81	0.6251	0.4926	0.0696	0.0260*	
H71	0.6559	0.5360	−0.0684	0.0289*	
H61	0.7301	0.7280	−0.1055	0.0281*	
H51	0.7718	0.8866	−0.0041	0.0283*	
H41	0.7470	0.8439	0.1359	0.0224*	
H91	1.0307	0.3132	0.4569	0.0196*	
H151	1.2192	0.1755	0.4814	0.0193*	
H141	1.3600	0.0204	0.4383	0.0195*	
H171	1.4887	−0.2006	0.3096	0.0295*	
H172	1.5153	−0.0849	0.3703	0.0296*	
H173	1.3961	−0.1841	0.3835	0.0292*	
H111	1.0173	0.1922	0.2457	0.0203*	
H161	1.0275	−0.0232	0.0825	0.0339*	
H162	1.0336	0.1188	0.1071	0.0348*	
H163	0.9479	0.0253	0.1552	0.0344*	
H221	0.9453	0.3651	0.7838	0.0209*	
H222	0.8212	0.2696	0.7549	0.0213*	
H281	1.0106	0.3923	0.9334	0.0253*	
H271	0.9755	0.3524	1.0704	0.0279*	
H261	0.7688	0.2572	1.0983	0.0275*	
H251	0.5958	0.1972	0.9872	0.0282*	
H241	0.6357	0.2334	0.8499	0.0246*	
H291	0.4816	0.6724	0.5376	0.0190*	
H311	0.2919	0.8124	0.5145	0.0188*	
H321	0.1533	0.9669	0.5598	0.0183*	
H361	0.0297	1.1869	0.6942	0.0279*	
H362	0.1185	1.1706	0.6193	0.0275*	
H363	0.0006	1.0703	0.6330	0.0275*	
H351	0.4958	0.7869	0.7466	0.0208*	
H371	0.4800	0.9831	0.9202	0.0331*	
H372	0.5668	0.9527	0.8439	0.0323*	
H373	0.4801	0.8448	0.8807	0.0322*	
H1	0.6713	0.5556	0.5505	0.0500*	
H2	0.8546	0.4361	0.4487	0.0500*	

### Atomic displacement parameters (Å^2^)

**Table:**

	*U*^11^	*U*^22^	*U*^33^	*U*^12^	*U*^13^	*U*^23^
S1	0.01948 (19)	0.0154 (2)	0.01721 (19)	0.00548 (15)	0.00564 (14)	0.00046 (14)
C1	0.0153 (7)	0.0141 (8)	0.0163 (7)	−0.0008 (6)	0.0044 (5)	0.0002 (6)
S2	0.0203 (2)	0.0195 (2)	0.01605 (19)	0.00695 (15)	0.00537 (14)	0.00125 (15)
C2	0.0181 (7)	0.0188 (8)	0.0198 (8)	0.0078 (6)	0.0035 (6)	0.0031 (6)
C3	0.0130 (7)	0.0213 (8)	0.0180 (7)	0.0056 (6)	0.0012 (5)	0.0007 (6)
C8	0.0185 (7)	0.0202 (8)	0.0258 (8)	0.0040 (6)	0.0022 (6)	−0.0008 (7)
C7	0.0236 (8)	0.0294 (10)	0.0202 (8)	0.0079 (7)	0.0010 (6)	−0.0066 (7)
C6	0.0198 (8)	0.0365 (10)	0.0172 (8)	0.0089 (7)	0.0032 (6)	0.0032 (7)
C5	0.0206 (8)	0.0258 (9)	0.0258 (8)	0.0035 (7)	0.0042 (6)	0.0048 (7)
C4	0.0194 (7)	0.0196 (8)	0.0206 (8)	0.0037 (6)	0.0020 (6)	−0.0019 (6)
N1	0.0182 (6)	0.0142 (7)	0.0177 (6)	0.0033 (5)	0.0064 (5)	0.0000 (5)
N2	0.0178 (6)	0.0135 (7)	0.0213 (7)	0.0013 (5)	0.0072 (5)	0.0002 (5)
C9	0.0187 (7)	0.0147 (8)	0.0189 (7)	−0.0001 (6)	0.0059 (6)	0.0008 (6)
C10	0.0154 (7)	0.0133 (8)	0.0189 (7)	−0.0012 (6)	0.0059 (6)	0.0012 (6)
C15	0.0174 (7)	0.0161 (8)	0.0167 (7)	−0.0015 (6)	0.0034 (6)	−0.0004 (6)
C14	0.0138 (7)	0.0160 (8)	0.0180 (7)	0.0000 (6)	0.0020 (5)	0.0028 (6)
C13	0.0142 (7)	0.0128 (7)	0.0201 (7)	0.0009 (6)	0.0059 (6)	0.0015 (6)
O2	0.0195 (5)	0.0177 (6)	0.0191 (5)	0.0067 (4)	0.0032 (4)	−0.0014 (4)
C17	0.0189 (8)	0.0195 (8)	0.0238 (8)	0.0068 (6)	0.0049 (6)	0.0032 (6)
C12	0.0176 (7)	0.0176 (8)	0.0156 (7)	−0.0004 (6)	0.0038 (6)	0.0003 (6)
C11	0.0159 (7)	0.0160 (8)	0.0190 (7)	0.0018 (6)	0.0027 (6)	0.0040 (6)
O1	0.0255 (6)	0.0276 (7)	0.0170 (5)	0.0088 (5)	0.0000 (4)	−0.0037 (5)
C16	0.0241 (8)	0.0318 (10)	0.0162 (8)	0.0025 (7)	−0.0003 (6)	0.0013 (7)
S21	0.01906 (19)	0.0154 (2)	0.01649 (19)	0.00483 (15)	0.00539 (14)	−0.00063 (14)
C21	0.0168 (7)	0.0135 (8)	0.0163 (7)	−0.0011 (6)	0.0058 (6)	−0.0015 (6)
S22	0.0232 (2)	0.0181 (2)	0.01609 (19)	0.00641 (16)	0.00685 (14)	0.00131 (15)
C22	0.0188 (7)	0.0184 (8)	0.0194 (8)	0.0056 (6)	0.0048 (6)	0.0033 (6)
C23	0.0195 (7)	0.0150 (8)	0.0194 (8)	0.0056 (6)	0.0049 (6)	0.0028 (6)
C28	0.0182 (8)	0.0207 (9)	0.0265 (8)	0.0040 (6)	0.0021 (6)	0.0019 (7)
C27	0.0250 (8)	0.0244 (9)	0.0230 (8)	0.0058 (7)	−0.0020 (6)	0.0001 (7)
C26	0.0309 (9)	0.0240 (9)	0.0181 (8)	0.0073 (7)	0.0048 (7)	0.0044 (7)
C25	0.0253 (8)	0.0245 (9)	0.0236 (8)	−0.0004 (7)	0.0055 (7)	0.0035 (7)
C24	0.0215 (8)	0.0234 (9)	0.0208 (8)	0.0004 (7)	0.0023 (6)	0.0009 (6)
N21	0.0193 (6)	0.0153 (7)	0.0166 (6)	0.0037 (5)	0.0069 (5)	−0.0002 (5)
N22	0.0189 (6)	0.0134 (7)	0.0217 (7)	0.0019 (5)	0.0090 (5)	0.0001 (5)
C29	0.0178 (7)	0.0157 (8)	0.0168 (7)	−0.0023 (6)	0.0056 (6)	−0.0007 (6)
C30	0.0152 (7)	0.0124 (7)	0.0202 (7)	−0.0007 (6)	0.0067 (6)	0.0014 (6)
C31	0.0176 (7)	0.0158 (8)	0.0148 (7)	−0.0020 (6)	0.0031 (6)	−0.0016 (6)
C32	0.0142 (7)	0.0169 (8)	0.0172 (7)	0.0010 (6)	0.0014 (5)	0.0027 (6)
C33	0.0148 (7)	0.0138 (8)	0.0184 (7)	0.0006 (6)	0.0047 (6)	0.0010 (6)
O21	0.0193 (5)	0.0178 (6)	0.0190 (5)	0.0068 (4)	0.0031 (4)	−0.0009 (4)
C36	0.0185 (7)	0.0173 (8)	0.0244 (8)	0.0066 (6)	0.0049 (6)	0.0038 (6)
C34	0.0185 (7)	0.0172 (8)	0.0146 (7)	−0.0001 (6)	0.0047 (6)	0.0007 (6)
C35	0.0166 (7)	0.0173 (8)	0.0171 (7)	0.0035 (6)	0.0024 (6)	0.0032 (6)
O22	0.0262 (6)	0.0276 (7)	0.0139 (5)	0.0110 (5)	0.0005 (4)	−0.0025 (5)
C37	0.0242 (8)	0.0298 (10)	0.0146 (7)	0.0055 (7)	0.0013 (6)	0.0013 (7)

### Geometric parameters (Å, º)

**Table:**

S1—C1	1.6807 (15)	S21—C21	1.6774 (15)
C1—S2	1.7494 (15)	C21—S22	1.7494 (15)
C1—N1	1.331 (2)	C21—N21	1.336 (2)
S2—C2	1.8244 (15)	S22—C22	1.8294 (16)
C2—C3	1.516 (2)	C22—C23	1.507 (2)
C2—H21	0.983	C22—H221	0.973
C2—H22	0.980	C22—H222	0.968
C3—C8	1.395 (2)	C23—C28	1.396 (2)
C3—C4	1.392 (2)	C23—C24	1.397 (2)
C8—C7	1.388 (2)	C28—C27	1.390 (2)
C8—H81	0.956	C28—H281	0.935
C7—C6	1.386 (3)	C27—C26	1.384 (3)
C7—H71	0.921	C27—H271	0.952
C6—C5	1.391 (3)	C26—C25	1.393 (2)
C6—H61	0.944	C26—H261	0.948
C5—C4	1.390 (2)	C25—C24	1.387 (2)
C5—H51	0.947	C25—H251	0.953
C4—H41	0.941	C24—H241	0.947
N1—N2	1.3845 (18)	N21—N22	1.3820 (18)
N1—H2	0.924	N21—H1	0.874
N2—C9	1.282 (2)	N22—C29	1.280 (2)
C9—C10	1.460 (2)	C29—C30	1.462 (2)
C9—H91	0.945	C29—H291	0.932
C10—C15	1.393 (2)	C30—C31	1.392 (2)
C10—C11	1.407 (2)	C30—C35	1.406 (2)
C15—C14	1.396 (2)	C31—C32	1.399 (2)
C15—H151	0.951	C31—H311	0.948
C14—C13	1.383 (2)	C32—C33	1.379 (2)
C14—H141	0.948	C32—H321	0.944
C13—O2	1.3612 (18)	C33—O21	1.3628 (18)
C13—C12	1.421 (2)	C33—C34	1.422 (2)
O2—C17	1.4325 (18)	O21—C36	1.4362 (18)
C17—H171	0.961	C36—H361	0.963
C17—H172	0.964	C36—H362	0.965
C17—H173	0.979	C36—H363	0.965
C12—C11	1.381 (2)	C34—C35	1.377 (2)
C12—O1	1.3603 (19)	C34—O22	1.3592 (19)
C11—H111	0.938	C35—H351	0.927
O1—C16	1.4273 (19)	O22—C37	1.4272 (18)
C16—H161	0.943	C37—H371	0.972
C16—H162	0.968	C37—H372	0.969
C16—H163	0.970	C37—H373	0.976
			
S1—C1—S2	125.49 (9)	S21—C21—S22	124.90 (9)
S1—C1—N1	120.52 (11)	S21—C21—N21	120.52 (11)
S2—C1—N1	113.98 (11)	S22—C21—N21	114.57 (11)
C1—S2—C2	102.05 (7)	C21—S22—C22	101.97 (7)
S2—C2—C3	107.65 (10)	S22—C22—C23	107.26 (10)
S2—C2—H21	110.3	S22—C22—H221	109.5
C3—C2—H21	109.7	C23—C22—H221	112.0
S2—C2—H22	107.9	S22—C22—H222	109.4
C3—C2—H22	110.5	C23—C22—H222	109.2
H21—C2—H22	110.7	H221—C22—H222	109.4
C2—C3—C8	121.01 (15)	C22—C23—C28	121.20 (14)
C2—C3—C4	119.67 (15)	C22—C23—C24	119.74 (15)
C8—C3—C4	119.32 (15)	C28—C23—C24	119.05 (14)
C3—C8—C7	120.44 (16)	C23—C28—C27	120.27 (15)
C3—C8—H81	119.0	C23—C28—H281	119.7
C7—C8—H81	120.5	C27—C28—H281	120.1
C8—C7—C6	120.09 (16)	C28—C27—C26	120.38 (16)
C8—C7—H71	120.5	C28—C27—H271	119.6
C6—C7—H71	119.5	C26—C27—H271	120.0
C7—C6—C5	119.73 (15)	C27—C26—C25	119.76 (15)
C7—C6—H61	118.7	C27—C26—H261	119.1
C5—C6—H61	121.5	C25—C26—H261	121.2
C6—C5—C4	120.34 (16)	C26—C25—C24	120.09 (16)
C6—C5—H51	119.1	C26—C25—H251	120.4
C4—C5—H51	120.6	C24—C25—H251	119.5
C3—C4—C5	120.06 (16)	C23—C24—C25	120.44 (16)
C3—C4—H41	118.9	C23—C24—H241	119.1
C5—C4—H41	121.1	C25—C24—H241	120.4
C1—N1—N2	120.71 (13)	C21—N21—N22	120.85 (12)
C1—N1—H2	118.7	C21—N21—H1	116.4
N2—N1—H2	120.6	N22—N21—H1	121.9
N1—N2—C9	113.83 (13)	N21—N22—C29	114.27 (13)
N2—C9—C10	122.28 (14)	N22—C29—C30	121.69 (14)
N2—C9—H91	120.4	N22—C29—H291	120.7
C10—C9—H91	117.3	C30—C29—H291	117.6
C9—C10—C15	117.98 (14)	C29—C30—C31	119.12 (14)
C9—C10—C11	122.42 (14)	C29—C30—C35	121.40 (14)
C15—C10—C11	119.58 (14)	C31—C30—C35	119.45 (14)
C10—C15—C14	120.50 (14)	C30—C31—C32	120.54 (14)
C10—C15—H151	120.0	C30—C31—H311	119.1
C14—C15—H151	119.5	C32—C31—H311	120.4
C15—C14—C13	120.08 (14)	C31—C32—C33	120.09 (14)
C15—C14—H141	119.4	C31—C32—H321	118.2
C13—C14—H141	120.5	C33—C32—H321	121.7
C14—C13—O2	125.39 (14)	C32—C33—O21	125.73 (13)
C14—C13—C12	119.77 (14)	C32—C33—C34	119.65 (14)
O2—C13—C12	114.83 (13)	O21—C33—C34	114.59 (13)
C13—O2—C17	117.10 (12)	C33—O21—C36	117.04 (12)
O2—C17—H171	106.1	O21—C36—H361	105.8
O2—C17—H172	111.9	O21—C36—H362	108.9
H171—C17—H172	109.6	H361—C36—H362	109.9
O2—C17—H173	109.3	O21—C36—H363	110.1
H171—C17—H173	109.9	H361—C36—H363	110.9
H172—C17—H173	110.0	H362—C36—H363	111.1
C13—C12—C11	119.89 (14)	C33—C34—C35	120.06 (14)
C13—C12—O1	114.08 (14)	C33—C34—O22	114.49 (13)
C11—C12—O1	126.01 (14)	C35—C34—O22	125.43 (14)
C10—C11—C12	120.16 (14)	C30—C35—C34	120.20 (14)
C10—C11—H111	120.7	C30—C35—H351	119.0
C12—C11—H111	119.1	C34—C35—H351	120.8
C12—O1—C16	117.16 (12)	C34—O22—C37	116.40 (12)
O1—C16—H161	106.2	O22—C37—H371	104.2
O1—C16—H162	110.2	O22—C37—H372	110.0
H161—C16—H162	111.1	H371—C37—H372	111.9
O1—C16—H163	110.8	O22—C37—H373	109.3
H161—C16—H163	110.4	H371—C37—H373	112.0
H162—C16—H163	108.1	H372—C37—H373	109.2

### Hydrogen-bond geometry (Å, º)

Cg1, Cg2 and Cg4 are the centroids of the C3–C8, C10–C15 and C30–C35 rings, respectively.

**Table:**

*D*—H···*A*	*D*—H	H···*A*	*D*···*A*	*D*—H···*A*
N1—H2···S21	0.92	2.52	3.418 (1)	166
N21—H1···S1	0.87	2.55	3.407 (1)	168
C16—H163···O21^i^	0.97	2.69	3.560 (2)	149
C17—H172···*Cg*4^ii^	0.97	2.74	3.5587 (18)	143
C28—H281···*Cg*1^ii^	0.94	2.94	3.6082 (18)	130
C36—H363···*Cg*2^i^	0.97	2.67	3.5023 (17)	145

Symmetry codes: (i) −*x*+1, −*y*+1, −*z*+1; (ii) −*x*+2, −*y*+1, −*z*+1.

## References

[bb1] Betteridge, P. W., Carruthers, J. R., Cooper, R. I., Prout, K. & Watkin, D. J. (2003). *J. Appl. Cryst.* **36**, 1487.

[bb2] Break, M. K. bin, Tahir, M. I. M., Crouse, K. A. & Khoo, T.-J. (2013). *Bioinorganic Chemistry and Applications*, Vol. 2013, Article ID 362513, 13 pages. http//dx..org/10.1155/2013/362513 10.1155/2013/362513 PMC3844202 24319401

[bb3] Fan, Z., Huang, Y.-L., Wang, Z., Guo, H.-Q. & Shan, S. (2011). *Acta Cryst.* E**67**, o3011. 10.1107/S1600536811042140 PMC3247410 22220028

[bb4] Groom, C. R. & Allen, F. H. (2014). *Angew. Chem. Int. Ed.* **53**, 662–671. 10.1002/anie.201306438 24382699

[bb5] Khoo, T.-J., Break, M. K., bin, , Crouse, K. A., Tahir, M. I. M., Ali, A. M., Cowley, A. R., Watkin, D. & Tarafder, M. T. H. (2014). *Inorg. Chim. Acta*, **413**, 68–76.

[bb6] Macrae, C. F., Bruno, I. J., Chisholm, J. A., Edgington, P. R., McCabe, P., Pidcock, E., Rodriguez-Monge, L., Taylor, R., van de Streek, J. & Wood, P. A. (2008). *J. Appl. Cryst.* **41**, 466–470.

[bb7] Oxford Diffraction (2002). *CrysAlis CCD* and *CrysAlis RED*. Oxford Diffraction Ltd, Abingdon, England.

[bb8] Palatinus, L. & Chapuis, G. (2007). *J. Appl. Cryst.* **40**, 786–790.

[bb9] Westrip, S. P. (2010). *J. Appl. Cryst.* **43**, 920–925.

